# Giant Pancreatic Pseudocyst after Coronary Artery Bypass Graft in a Hemodialysis Patient: A Case Report

**DOI:** 10.3390/clinpract13050111

**Published:** 2023-10-08

**Authors:** Ming-Jen Chan, Chun-Yih Hsieh, Yi-Jiun Su, Chien-Chang Huang, Wen-Hung Huang, Cheng-Hao Weng, Tzung-Hai Yen, Ching-Wei Hsu

**Affiliations:** 1 Department of Nephrology, Clinical Poison Center, Chang Gung Memorial Hospital, Linkou Medical Center, Taoyuan City 333423, Taiwan; b9202066@cgmh.org.tw (M.-J.C.); m7199@adm.cgmh.org.tw (C.-Y.H.); hccchris@gmail.com (C.-C.H.); williammedia@yahoo.com.tw (W.-H.H.); drweng@seed.net.tw (C.-H.W.); m19570@adm.cgmh.org.tw (T.-H.Y.); 2 School of Medicine, College of Medicine, Chang Gung University, Taoyuan City 333323, Taiwan; 3 Graduate Institute of Clinical Medical Science, College of Medicine, Chang Gung University, Taoyuan City 333323, Taiwan; 4 Division of Hematology-Oncology, Department of Internal Medicine, Linkou Chang Gung Memorial Hospital, Taoyuan City 333423, Taiwan; b9305038@cgmh.org.tw

**Keywords:** cardiopulmonary bypass, coronary artery bypass graft, end-stage renal disease, hemodialysis, pancreatitis, pseudocyst

## Abstract

End-stage renal disease (ESRD) patients have a high prevalence of coronary artery disease, and coronary artery bypass graft (CABG) is one of the essential treatments. ESRD patients undergoing CABG surgery have an increased risk of postoperative complications, including acute pancreatitis. Here, we present the unique case of an exceptionally large pancreatic pseudocyst caused by pancreatitis in an ESRD patient after CABG surgery. A 45-year-old male with ESRD under maintenance hemodialysis received CABG surgery for significant coronary artery disease. Two weeks later, he experienced worsening abdominal pain and a palpable mass was noticed in the epigastric region. Computer tomography revealed an unusually large pseudocyst measuring 21 × 17 cm in the retroperitoneum due to necrotizing pancreatitis. The patient underwent percutaneous cystic drainage, and the symptoms were significantly improved without surgical intervention. Factors such as prolonged cardiopulmonary bypass time, postoperative hypotension, and intradialytic hypotension appeared to have contributed to the development of severe pancreatitis in this case. This report highlights the rarity of a giant pancreatic pseudocyst in an ESRD patient after CABG surgery and emphasizes the importance of vigilant postoperative care.

## 1. Introduction

End-stage renal disease (ESRD) patients have a high prevalence of coronary artery disease, and their cardiovascular mortality rates are almost 20 times higher than those in the general population [1]. The high prevalence of coronary artery disease in ESRD patients is significantly influenced by the presence of comorbidities such as hypertension, diabetes mellitus, dyslipidemia, and tobacco use [2]. Additionally, in ESRD patients, several factors including uremia, inflammatory responses, elevated oxidative stress, neurohormonal activation, and endothelial dysfunction can contribute to the progression of coronary artery disease and the formation of atherosclerosis [2]. Although the optimal modality of revascularization therapy remains controversial in ESRD patients, coronary artery bypass graft (CABG) has been recommended as a treatment for significant coronary artery disease in these patients [3]. However, ESRD patients undergoing CABG are at an increased risk of postoperative mortality and complications compared to patients without kidney disease [4]. In a regional prospective cohort study of 15,500 consecutive patients who underwent CABG in northern New England from 1992 to 1997, individuals with end-stage renal disease (ESRD) were found to be 3.1 times more likely to experience post-CABG mortality than those with normal renal function (95% confidence interval 2.1 to 4.7) [4]. Additionally, the study also reported that ESRD patients had a higher risk of postoperative mediastinitis and postoperative stroke. Moreover, CABG causes a significant financial burden on ESRD patients, ranking among the top five non-dialytic medical expenditures [5]. Hence, optimizing perioperative care for ESRD patients undergoing CABG is crucial. 

CABG is associated with several major complications, such as acute myocardial infarction, stroke, wound infection, prolonged mechanical ventilation, acute kidney injury, postoperative hemorrhage, and even death [6]. Among these complications, gastrointestinal complications are not commonly observed, but the mortality rate can be as high as 33% [7]. Gastrointestinal complications not only impact the gastrointestinal system but also increase the likelihood of encountering other adverse postoperative outcomes [7]. Patients with gastrointestinal complications have an increased risk of stroke, myocardial infarction, sternal wound infection, the need for reoperation due to bleeding, renal failure, and respiratory failure [7]. The gastrointestinal complications after CABG include abscess, ileus, and gastrointestinal ulcers with bleeding [8]. Of these, severe acute pancreatitis has been reported to develop in approximately one percent of post-CABG patients [9]. The primary risk factors for pancreatic cellular injury are preoperative renal dysfunction, perioperative calcium administration, and postoperative hypotension [9]. However, the diagnosis of acute pancreatitis in post-CABG patients with kidney dysfunction can be a challenge due to the falsely increased serum level of amylase [10].

Here, we present a case of necrotizing pancreatitis complicated with a giant pseudocyst in an ESRD patient after CABG surgery. Only a few cases of giant pancreatic pseudocysts in this population have been documented to date.

## 2. Case Report

A 45-year-old male had ESRD caused by chronic glomerulonephritis. He had been undergoing 4 h of hemodialysis with a blood flow of 250 mL/min through an arteriovenous fistula three times a week for 11 years. The hemodialysis was conducted using single-use hollow-fiber dialyzers with polysulfone membranes. Three years ago, he received a total parathyroidectomy with auto-transplantation over the left arm due to secondary hyperparathyroidism. The patient took a daily dosage of 6003 mg of calcium acetate in conjunction with meals to manage phosphate levels. He did not have the habits of smoking or alcohol consumption, but did have a history of dyslipidemia and coronary artery disease, which were adequately controlled with medication. However, he had recently visited our emergency department several times because of intermittent chest pain and diaphoresis. He underwent a series of studies, including cardiac catheterization. The coronary angiography revealed triple-vessel disease with left main coronary artery stenosis. Notably, there was a critical stenosis of 82% from the left main branch to the left anterior descending coronary artery ostium. Because of this, he underwent conventional CABG surgery two months ago.

During the CABG surgery, the patient was on cardiopulmonary bypass for a total of 286 min, with aortic clamping for 54 min. Intraoperatively, he received four units of packed red blood cell transfusion. During the operation, a bypass procedure was carried out, involving the left internal thoracic artery connecting to the left anterior descending artery, as well as a free graft using the right internal thoracic artery to the third obtuse marginal artery. After the surgery, the patient received norepinephrine infusion to stabilize blood pressure, with a maximum dosage of 0.11 mcg/kg/min. He was successfully weaned off the inotropic agent within one day of the operation. The postoperative ejection fraction of the left ventricle, measured by echocardiogram, was 58%.

**Figure 1:**
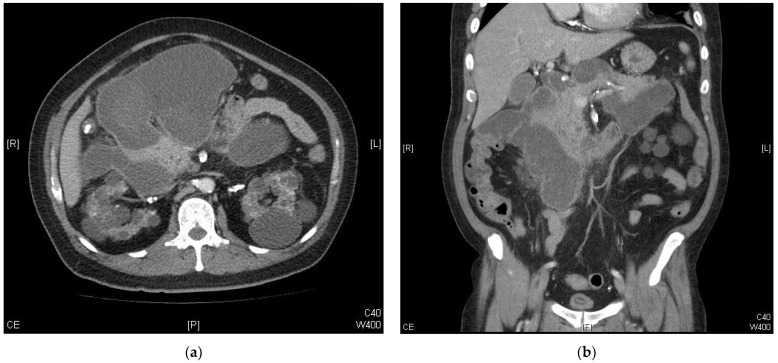
Contrast-enhanced abdominal computed-tomography images of the patient: (**a**) the axial plane and (**b**) the coronal plane.

Following his CABG surgery, the patient experienced intermittent epigastric pain but was discharged after recovering from the procedure. The pain was dull and occasionally radiated to his back. It was aggravated by eating and improved during fasting. The pain could be triggered occasionally during dialysis session, particularly when intradialytic hypotension occurred with a higher ultrafiltration rate (14.86 mL/kg/h). In addition to the clinical symptoms, a non-tender bulge had developed in his epigastric area with progressive enlargement over a period of about 2 weeks. He was readmitted due to these symptoms and the enlarged mass lesion. Physical examination disclosed a large and firm mass in the upper abdomen. The mass was round in shape, well-demarcated with the sensation of being fluid-filled. There were no apparent skin discolorations over the affected area. The laboratory data showed increased amylase and lipase (Table 1). A contrast-enhanced abdominal computed tomography (CT) scan revealed a giant multiloculated cystic lesion with a size of 21 × 17 cm in the retroperitoneum surrounding the pancreas and duodenum. This finding was consistent with necrotizing pancreatitis and pseudocyst formation (Figure 1). The CT scan did not reveal any biliary structure abnormality.

**Figure 2:**
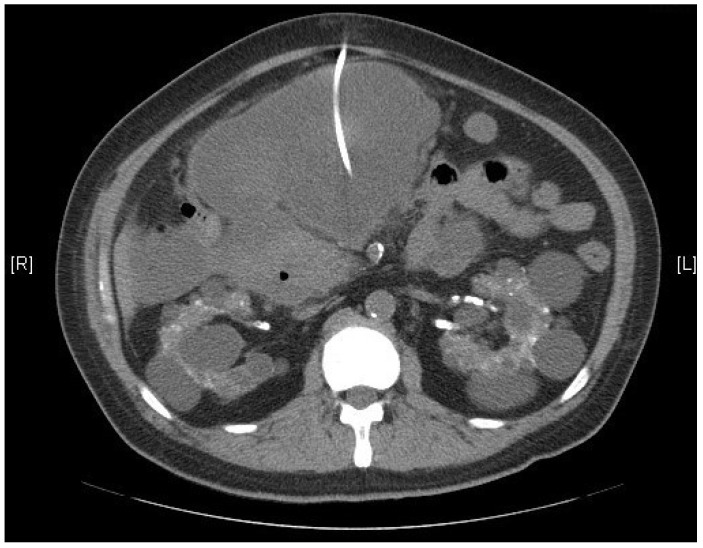
Computed-tomography-guided drainage for pancreatic pseudocyst.

Following the diagnosis, CT-guided cystic drainage was performed successfully (Figure 2), and the amylase level in the cystic fluid was measured at 10,186 U/L. This elevated amylase level confirms the pancreas as the origin of the pseudocyst. The patient did not experience dyspnea during the following two days, and follow-up laboratory data are summarized in the Table 1. The clinical symptoms were significantly improved after cystic drainage and supportive treatment, and surgical intervention was not required. The patient was discharged from the hospital and has received maintenance hemodialysis in our dialysis units.

## 3. Discussion

There are many potential complications in patients receiving CABG surgery [11]. Among them, gastrointestinal complications are particularly critical due to their high mortality rate [7]. Acute pancreatitis after cardiac surgery has been reported in previous literature (Table 2). However, acute pancreatitis complicated with giant pseudocysts is rarely reported in patients after CABG surgery. In a study of 5621 patients receiving cardiopulmonary bypass (CPB), 25 patients developed acute pancreatitis [12]. Among them, 10 had pancreatic necrosis, resulting in 11 deaths, which equated to a mortality rate of 44% when acute pancreatitis occurred [12]. However, there were no reported cases of pancreatic pseudocyst development, highlighting the rarity of pseudocyst formation in this clinical context. The currently prevailing hypothesis regarding the cause of postoperative acute pancreatitis is related to splanchnic ischemia [9,13]. Low cardiac output in patients with cardiac surgery receiving CPB can lead to pancreatic injury due to splanchnic ischemia [13]. Elevated intracellular and intramitochondrial calcium concentrations are observed during ischemia [13]. Ischemia potentially increases intra-acinar calcium levels, triggering intracellular trypsinogen activation and the recruitment of neutrophils into the pancreatic circulation. These processes may ultimately lead to the development of acute pancreatitis [13]. The duration of CPB was observed to be correlated with pancreatic injury, as patients with pancreatic cellular injury tended to have longer CPB duration compared to those without [9]. Notably, our patient underwent a significantly prolonged CPB time of 286 min with aortic clamping, surpassing the average CPB time (124 ± 44.8 min) documented for individuals with pancreatic injury in the aforementioned study [9]. In addition to CPB-related ischemia, the blood lost during the operation and the occurrence of postoperative shock with the requirement of inotropic agents may further worsen splanchnic ischemia. In the porcine septic shock model, norepinephrine redirects blood flow away from the mesenteric circulation and decreases blood flow in the pancreas [14]. The combination of aforementioned factors may contribute to splanchnic ischemia, leading to the development of necrotizing pancreatitis complicated by the formation of an unusual giant pseudocyst in this patient.

**Figure 3:**
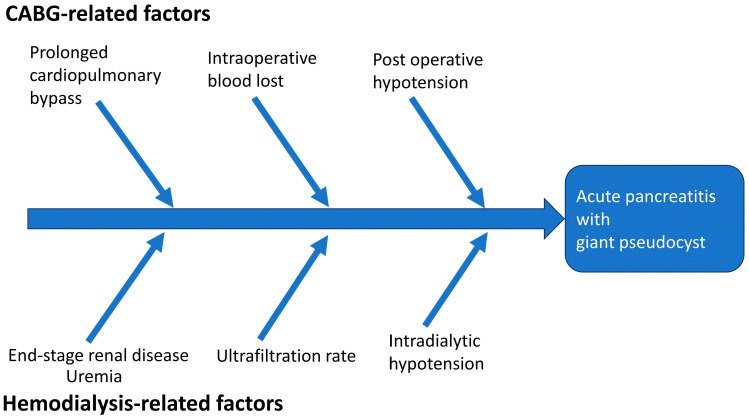
Proposed contributing factors for acute pancreatitis with pseudocyst formation in our patient. Please note that the proportion of each contribution may not have been equal.

In addition to perioperative factors, several other factors may play a role in the development of acute pancreatitis with the formation of a giant pseudocyst (Figure 3). Among these factors, individuals with ESRD have a higher incidence of acute pancreatitis and adverse events after CABG compared to those without ESRD [4,21]. There are several possible mechanisms proposed to explain why ESRD patients are more susceptible to acute pancreatitis. A higher level of uremic toxins is linked to an increased risk of pancreatitis [21]. The precise mechanism of uremia leading to pancreatitis is still under investigation. A study reported that elevated serum levels of cholecystokinin, glucagon, and gastric inhibitory polypeptide in patients with ESRD may lead to the overproduction of pancreatic enzymes, potentially resulting in impaired pancreatic function and pancreatic abnormalities [22]. Intradialytic hypotension and a higher ultrafiltration rate during hemodialysis may further compromise the already vulnerable splanchnic circulation [23]. A previous study of 91 hemodialysis patients demonstrated that decreased hepato-splanchnic circulation saturation was correlated with systemic systolic blood pressure [24]. Interestingly, this reduction in saturation was more pronounced among patients with intradialytic hypotension when compared to those without (−13.8 ± 9.3% vs. 0.4 ± 9.8%, *p* < 0.001). The decline in hepato-splanchnic circulation saturation was found to be associated with the percentage changes in systolic blood pressure and the ultrafiltration. In addition, there is evidence that ESRD patients undergoing peritoneal dialysis may have a higher risk of developing pancreatitis compared to those undergoing hemodialysis [25]. Peritoneal dialysis patients have a higher occurrence of abdominal anatomical anomalies, and the risk of pancreatic injury is further elevated due to the presence of toxic substances from the peritoneal dialysate, bags, or tubing [25].

A higher serum calcium level and hyperparathyroidism may predispose patients to the deposition of calcium within the pancreatic duct and trigger the occurrence of pancreatitis [26]. Hypercalcemia can be induced by various factors, including hyperparathyroidism, certain medications, and even intravenous calcium administration, all of which have been documented as potential causes of acute pancreatitis [27]. Although the patient had received a parathyroidectomy previously, the serum levels of calcium were approaching the upper limit of normal range due to the chronic use of a high daily dosage of calcium-based phosphate binders. Secondary hyperparathyroidism in ESRD patients causes unique metabolic abnormalities and pathophysiology distinct from those observed in primary hyperparathyroidism [28]. Despite the high cure rate of acute pancreatitis by parathyroidectomy in patients with primary hyperparathyroidism, there is currently no available literature that examines whether treating secondary hyperparathyroidism can reduce the risk of acute pancreatitis in ESRD patients [29,30]. In exceptional situations like pica, the use of calcium acetate could potentially lead to hypercalcemia and necrotizing pancreatitis [31]. Whether this oral calcium intake can trigger pancreatitis in the absence of hypercalcemia requires further investigation. Further research is needed to clarify the potential association between treated secondary hyperparathyroidism and the occurrence of acute pancreatitis. 

Considering the combination of contributing factors, this patient may be in the situation of a “perfect storm” for the development of acute pancreatitis. For this patient, acute pancreatitis following CABG appears to have been influenced by several factors. These include splanchnic ischemia caused by prolonged cardiopulmonary bypass, intraoperative blood loss, postoperative hypotension, a high ultrafiltration rate during dialysis, and episodes of intradialytic hypotension. Additionally, the patient’s medical history of ESRD may have rendered him more susceptible to the development of acute pancreatitis. The occurrence of a giant pancreatic pseudocyst in an ESRD patient after CABG surgery is exceptionally rare and noteworthy. This unique case emphasizes the utmost importance of vigilant monitoring and comprehensive postoperative care for ESRD patients receiving CABG. Prompt identification and management of any unusual complications is crucial to achieving the best outcomes for these patients.

## Figures and Tables

**Table 1:** Patient’s laboratory examination result.

Lab Finding	at Admission	Two Days Later	Reference Range
WBC (/uL)	8000	7600	3900–10,600
Hemoglobin (g/dL)	9.4	9.4	13.5–17.5
Hematocrit (%)	31.6	31.6	41–53
Amylase (U/L)	387	86	28–100
Lipase (U/L)	1048	99	11–82
AST (U/L)	24		13–40
Glucose (mg/dL)	117		74–100
LDH (U/L)	414		135–260
Calcium (mg/dL)	9.5	10.2	8.6–10.3
Triglyceride (mg/dL)	168		<150
BUN (mg/dL)	33.5	41	7–25
Albumin (g/dL)		3.07	3.5–5.4
Intact-PTH (pg/mL)	49.5		15–65

Abbreviations: AST: aspartate aminotransferase; BUN: post-dialytic blood urea nitrogen; LDH: lactate dehydrogenase; PTH: parathyroid hormone; WBC: white blood cell.

**Table 2:** Notable studies reporting acute pancreatitis after cardiac surgery.

Study	Year	Country	Operation Type	Number	Incidence of AP	Note
Ohno et al. [15]	2023	Japan	Aortic arch surgery	353	4% (*n* = 14)	None required drainage
Elgharably et al. [16]	2021	US	All cardiac surgery	29,909	0.13% (*n =* 38)	
Marsoner et al. [17]	2019	Austria	On-pump cardiac surgery	4883	0.84% (*n =* 41)	Longer on-pump time in patients with GI complications (OR 1.006, 95%CI 1.001–1.011)
Musleh et al. [18]	2003	UK	CABG (on- and off-pump)	2327	0.13% (*n =* 3)	Off-pump and on-pump techniques had similar rates of GI complications
Fitzgerald et al. [19]	2000	US	All cardiac surgery	14,521	0.19% (*n =* 27)	More visceral ischemia with longer pump times and ESRD patients
Yilmaz et al. [20]	1996	Turkey	All cardiac surgery	3158	0.63% (*n =* 2)	
Lefor et al. [12]	1992	US	CABG	5621	0.44% (*n =* 25)	None had pancreatic pseudocysts
Castillo et al. [9]	1991	US	Cardiac surgery patients with CPB	300	7.7% (*n =* 23)	

Abbreviations: AP: acute pancreatitis; CABG: coronary artery bypass surgery; CI: confidence interval; CPB: cardiopulmonary bypass; ESRD, end-stage renal disease; GI: gastrointestinal; OR: odds ratio; UK: United Kingdom; US: United States.

## Data Availability

The data presented in this study are available on reasonable request from the corresponding author. The data are not publicly available due to patient privacy.
